# The effect of tapered flow resistive loading inspiratory muscle training on respiratory function in post-stroke tracheostomy patients: study protocol for a parallel-group, assessor-blinded randomized controlled trial

**DOI:** 10.3389/fneur.2025.1625289

**Published:** 2025-09-03

**Authors:** Fuqiang Wang, Yaojiang Li, Yunhong Deng, Congping Huang, Xiaodi Li, Kui Fan, Lixia Deng, Xiao Lv

**Affiliations:** ^1^Department of Rehabilitation Medicine, Guangdong Sanjiu Brain Hospital, Guangzhou, China; ^2^Department of Rehabilitation Therapy, Guangdong Sanjiu Brain Hospital, Guangzhou, China; ^3^Department of Rehabilitation Medicine, The First People’s Hospital of Tianmen, Tianmen, China

**Keywords:** stroke, tracheostomy, tapered flow resistive loading, maximum inspiratory pressure, inspiratory muscle training, decannulation success rate

## Abstract

**Background:**

Post-stroke tracheostomy patients frequently exhibit diverse levels of respiratory dysfunction. Inspiratory muscle training has demonstrated efficacy as an intervention to enhance respiratory function in these patients. However, conventional methods of inspiratory muscle training often fall short in terms of load regulation and individual adaptability. Tapered Flow Resistive Loading Inspiratory Muscle Training (TFRL-IMT) represents an innovative training modality that offers distinct advantages in augmenting respiratory muscle function. Nonetheless, its application in post-stroke tracheostomy patients remains under-researched, necessitating further systematic investigation to ascertain its clinical value.

**Methods and analysis:**

This investigation will employ a single-center, assessor-blinded, parallel-group randomized controlled trial design, enrolling 60 post-stroke tracheostomy patients (planned age range 18–70 years; and gender distribution will be collected and analyzed). Stratified compartmental group randomization will be utilized to allocate participants to either the experimental (*n* = 30) or control group (*n* = 30) in a 1:1 ratio. Both groups will receive conventional treatment, while the experimental group will additionally undergo TFRL-IMT using an electronic device that provides inspiratory resistance which dynamically decreases with increasing lung volume for a duration of three weeks. The primary outcome measure will be the rate of successful decannulation, with secondary outcomes encompassing diaphragm function, respiratory parameters, clinical outcomes, and quality of life assessments. The primary outcome (decannulation success rate) will be compared between groups using the Chi-square test.

**Discussion:**

TFRL-IMT may enhance respiratory function in patients through several mechanisms, including the provision of dynamic loading that aligns with the pressure-volume relationship of the respiratory muscles, the facilitation of neuromuscular adaptive changes, the optimization of the oxidative capacity of respiratory muscle fibers, and the remodeling of the central control pattern of the respiratory muscles. Nonetheless, the current study is subject to certain limitations, including its single-center design, a relatively short follow-up period, and some degree of device dependence and a high degree of heterogeneity in the stroke patient population.

**Clinical trial registration:**

https://www.chictr.org.cn/, identifier ChiCTR2500097604.

## Introduction

Stroke is one of the leading cause of disability and mortality worldwide. According to the Global Burden of Disease Study (GBD) 2021 data, there are approximately 11.9 million new cases of stroke globally each year, with a total prevalence reaching up to 93.8 million (1). In the clinical management of acute stroke, about 10% of patients require endotracheal intubation and mechanical ventilation support (2). As treatment advances, 15–35% of these patients necessitate a tracheotomy to sustain long-term ventilatory support due to delayed recovery of respiratory function (3). Even after successful weaning from mechanical ventilation, 26.5% of patients still require retention of the tracheal tube (4), and only 59% of stroke patients who undergo tracheotomy are successfully extubated within 12 months (5).

Post-stroke tracheostomy patients present significant challenges in their clinical management (6). These patients frequently manifest varying degrees of respiratory dysfunction, including reduced respiratory muscle strength (7), abnormal breathing patterns (8), impaired airway defense mechanisms (9), and diminished vital capacity (10). Although tracheotomy can effectively maintain airway patency, it also prolongs the patients’ hospital stay, escalates healthcare expenditures, and elevates mortality risk and complication rates, with common complications including pneumonia, airway stenosis, and ventilation-associated lung injury (11). From a pathophysiological perspective, prolonged tracheotomy status induces respiratory muscle dysfunction (8), which subsequently results in attenuated cough reflex and compromised airway clearance, and these factors interact with each other to form a vicious circle that impedes successful decannulation (9, 12). A systematic review demonstrated that successful decannulation in post-stroke tracheostomy patients was closely related to the recovery of respiratory muscle function, suggesting that improving respiratory muscle function may be one of the key factors to facilitate decannulation (13).

Inspiratory Muscle Training (IMT) represents a therapeutic intervention that utilizes resistance to enhance strength and endurance of respiratory musculature. Clinical evidence substantiates that IMT significantly enhances respiratory muscle performance in patient (14). Several studies have indicated that IMT enhances not only respiratory muscle strength but also coughing efficacy and accelerates pulmonary functional recovery (15, 16). Moodie et al. (17) conducted a systematic review validating the therapeutic efficacy of IMT for improving respiratory muscle function in mechanically ventilated patients, particularly among tracheostomized individuals with impaired respiratory muscle strength. Their analysis revealed that IMT significantly augments both maximum inspiratory pressure (MIP) and maximum expiratory pressure (MEP). Furthermore, a randomized controlled trial demonstrated improved MIP, higher success rates of ventilator weaning, and reduced hospitalization duration among mechanically ventilated patients undergoing IMT protocols (18). Studies in patients with neurological disorders indicate that regular IMT not only improves respiratory function, but also improves vital capacity and inspiratory reserve capacity, which is clinically important for the tracheotomy decannulation process (19).

Although IMT has achieved significant results in clinical practice, there are still many limitations of the conventional IMT method. Conventional threshold loading equipment is simple in structure and cannot dynamically adjust the loading intensity according to the different lung volume levels of patients, which leads to suboptimal training outcomes (20); Furthermore, conventional IMT mode lacks real-time feedback mechanisms and progress monitoring capabilities, which makes it difficult to maintain patient adherence and ensure the quality of training (21). Current IMT approaches are not individually designed to meet the different pathophysiological mechanisms, especially for patients with neuromuscular diseases, which do not fully consider their special respiratory dysfunction patterns, thus limiting the further improvement of clinical efficacy (22). Most conventional IMT adopts a fixed resistance design, which is not able to adapt to the dynamic demands of the whole respiratory cycle of the patient. This limitation is particularly pronounced during peak inspiratory phases, culminating in diminished training efficacy (23).

Tapered Flow Resistive Loading Inspiratory Muscle Training (TFRL-IMT) is a novel technique for strengthening inspiratory muscles. It delivers a flow resistance load that progressively decreases as lung volume increases, using an electronically controlled resistance valve system, which is designed to better match the pressure–volume characteristics of the respiratory muscles, thereby addressing the limitations of traditional training methods (24). Compared to conventional threshold-loading training, TFRL-IMT offers more uniform loading throughout the respiratory cycle, resulting in greater lung volume expansion and elevated inspiratory flow rates at equivalent training intensities, thereby increasing the total inspiratory workload (23). Modern TFRL-IMT devices incorporate electronic control systems with data storage capabilities, enabling the recording and monitoring of training parameters (25), a functional feature that helps to improve training quality and provides an objective basis for evaluation. Clinical studies have demonstrated that electronic IMT devices are feasible and safe for critically ill patients requiring prolonged mechanical ventilation, with high levels of patient acceptance (26). These findings constitute preliminary evidence supporting the application of TFRL-IMT in post-stroke tracheostomy populations.

Current research on TFRL-IMT has primarily focused on mechanically ventilated patients or individuals with chronic obstructive pulmonary disease (COPD), whereas studies involving post-stroke tracheostomized patients remain relatively scarce. Most existing studies on this population have concentrated on short-term improvements in physiological parameters, with limited systematic evaluation of long-term clinical outcomes. Therefore, further high-quality clinical trials are warranted to address this research gap and to elucidate the therapeutic potential of TFRL-IMT in post-stroke tracheostomized patients.

Therefore, the primary aim of this randomized controlled trial is to evaluate the effects of TFRL-IMT on respiratory function, respiratory muscle strength, decannulation rate, and health-related quality of life in post-stroke patients with tracheostomies. The study hypothesized that a structured TFRL-IMT program would be more effective than conventional respiratory rehabilitation methods in enhancing respiratory muscle strength, improving pulmonary function parameters, facilitating tracheostomy decannulation, and enhancing patients’ quality of life. This research is anticipated to offer a novel therapeutic approach for the clinical management of post-stroke tracheostomy patients and to establish a standardized and generalizable respiratory muscle training program, which is expected to significantly improve the long-term prognosis and functional recovery in this patient population.

## Methods and analysis

### Study design

**Figure 1:**
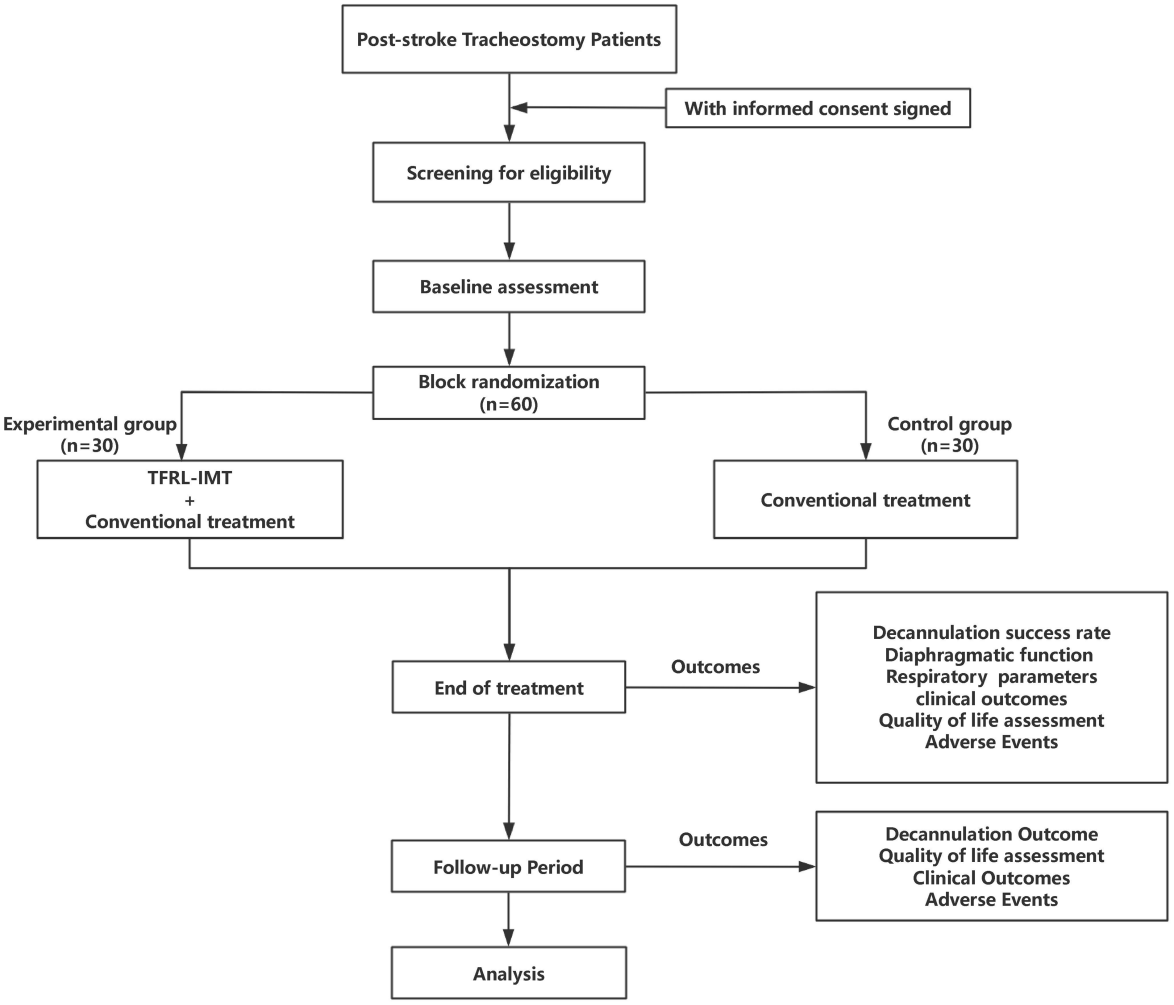
Trial flowchart. Diaphragmatic function assessment will include diaphragmatic mobility and diaphragmatic thickness. Respiratory function parameters will include MIP, FVC, PEF, and FEV1. Clinical outcome evaluation will include length of hospital stay, incidence of respiratory-related complications, and re-intubation rate within 72 h after decannulation. Quality of life assessment will include the EQ-5D-5L Health-Related Quality of Life Questionnaire, the Breathing Difficulty Assessment (Borg Scale), and the Hospital Anxiety and Depression Scale (HADS).

This study is a single-centre, evaluator-blinded, parallel-group randomised controlled trial aimed at evaluating the clinical efficacy of TFRL-IMT in post-stroke tracheostomized patients. The trial will be conducted at Guangdong Sanjiu Brain Hospital from March 2025 to February 2027. The detailed study procedures are illustrated in Figure 1.

### Participants and recruitment

This study is set to be conducted conducted within the Department of Rehabilitation Medicine at Guangdong Sanjiu Brain Hospital (Guangzhou, Guangdong, China), and will include a total of 60 post-stroke tracheostomy patients. The process for screening study participants will proceed as follows: first, newly admitted patients will be identified the hospital’s electronic medical record system. Rehabilitation physicians will then perform a preliminary evaluation of individuals who may be eligible. For those patients who preliminarily satisfy the inclusion criteria, researchers will engage in face-to-face discussions with them and their families to thoroughly explain the study’s objectives, methodology, and potential risks. Upon obtaining the patients’ consent to participate, researchers will undertake a comprehensive assessment to ensure that all inclusion criteria are met and that no exclusion criteria apply. Ultimately, eligible patients or their legal representatives will be required to sign a written informed consent form and will be formally enrolled in the study.

Baseline demographic and clinical characteristics, including age, gender distribution, stroke type (ischemic/hemorrhagic), and time since stroke, will be documented during enrollment and reported in subsequent outcome publications.

Inclusion criteria: (1) aged between 18 and 70 years; (2) first-evert or recurrent stroke (ischaemic or haemorrhagic) confirmed by cranial CT or MRI; (3) haemodynamically stable, defined as systolic blood pressure between 90–180 mmHg and diastolic pressure between 60–100 mmHg, without the need for vasoactive medication; (4) patients with stroke-related complications (e.g., impaired consciousness, dysphagia or respiratory failure) who underwent tracheotomy and remained clinically stable for ≥72 h post-procedure; (5) Glasgow Coma Scale score of ≥12, and the ability to comprehend and follow simple commands; (6) the patient or their legal representative is capable of understand the study protocol and provide written informed consent.

Exclusion criteria: (1) neuromuscular disorders (e.g., myasthenia gravis, amyotrophic lateral sclerosis) or primary thoracic deformity; (2) comorbid moderate-to-severe lung disease (e.g., COPD, interstitial lung fibrosis) or active pulmonary infection; (3) myocardial infarction, acute heart failure, or unstable cardiac arrhythmia within the past 3 months; (4) pneumothorax within the past month or presence of high-risk factors for pneumothorax; (5) use of medications that may impair neuromuscular function or induce drowsiness (e.g., high-dose sedatives, muscle relaxants); (6) participation in other interventional studies that may influence respiratory function during the same period; (7) expected survival of less than 3 months; (8) severe cognitive impairment that precludes completion of assessment or training procedures.

### Sample size calculation

The sample size calculation for this study was based on data obtained from a pre-trial conducted between October 2024 and January 2025. In the pre-trial, 24 post-stroke tracheostomy patients meeting the study’s inclusion and exclusion criteria were enrolled and randomly assigned to either the TFRL-IMT group (*n* = 12) or the conventional treatment group (*n* = 12). In the conventional treatment group, 6 patients were successfully extubated, resulting in a 50% decannulation success rate. In the TFRL-IMT group, 10 patients were extubated, with an decannulation success rate of 83.3%. The between-group difference in decannulation success rates was 33.3%. Based on the preliminary results and relevant literature (5), the decannulation success rate was conservatively estimated to be 50% in the control group and 80% in the TFRL-IMT group, yielding an expected effect size of 30%.

The sample size was calculated using PASS 15.0 software (NCSS, LLC, Kaysville, UT, United States). A significance level of *α* = 0.05 (two-sided) and statistical power (1 − *β*) = 80% were adopted based on literature recommendations (27). Assuming an decannulation success rate of 50% in the control group and 80% in the TFRL-IMT group, the required sample size for each group was calculated to be 26. Based on the preclinical follow-up experience of the research team, an expected attrition rate of 15% was applied. According to the adjustment formula *n*’ = *n*/(1 − f), where *n* is the unadjusted sample size and f is the expected attrition rate (28), the adjusted sample size per group was calculated as *n*’ = 26/(1–0.15) ≈ 30. Therefore, the finalised total sample size was determined to be 60.

### Randomisation and blinding

This study will adopt a stratified block randomized controlled design, with participants randomly assigned to the experimental or control group in a 1:1 ratio. Two stratification factors, age (≤40 years vs. >40 years) and stroke type (ischemic vs. hemorrhagic), will be incorporated into the randomization process to ensure balanced distribution of key prognostic variables between groups. The random allocation sequence will be generated using a computer-based random number generator, and group assignment will be conducted by an individual independent of the study team to ensure allocation concealment.

Owing to the nature of the intervention, blinding of participants and treatment providers will not be feasible. To minimise measurement bias, a strict single-blind design will be employed: all outcome assessments will be conducted by independent, standardised assessors who will remain blinded to group allocation; data entry will be performed by research assistants blinded to group allocation; and statistical analyses will be conducted independently by statisticians not involved in the research team. In addition, all research team members, except those directly administering the intervention, will remain blinded to group allocation throughout the trial.

### Intervention protocol

#### Experimental group interventions

In this study, patients in the experimental group will receive TFRL-IMT in addition to conventional treatment. TFRL-IMT will be administered using the inspiratory muscle training module of a portable pulmonary function testing device (X1, Seck Medical Devices Co., Ltd., Xiamen, China). The initial training load will be set at 30% of the subject’s MIP, which will be measured using the device’s built-in function. During the study period, MIP will be measured weekly, and the training load will be adjusted to 30% of the current MIP. Additionally, resistance will be gradually increased by 5% per week until 60% of MIP is reached and maintained, depending on the patient’s tolerance.

The training programme will last for 3 weeks, during which patients will undergo two sessions per day, each comprising five sets of six maximal inspiratory efforts with a one-minute rest between sets. During each session, the patient’s tracheal cannula will be connected to the device valve via a specialized connecting tube, and the patient will be instructed to inhale as forcefully and rapidly as possible, followed by slow exhalation. All training will be conducted under the supervision of healthcare professionals to ensure training quality and patient safety. The rehabilitation therapist will document the actual load, completion status, and any adverse events during each training session.

In patients with concurrent respiratory infections, appropriate antibiotics will be administered based on clinical manifestations and sputum culture results. In patients with thick sputum, nebulized salbutamol (2.5 mg) combined with ipratropium bromide (0.5 mg) will be administered three times daily at 8:00 a.m., 12:00 p.m., and 8:00 p.m.

To evaluate the efficacy and safety of the intervention, MIP values will be measured before and after training using the same portable pulmonary function device. Simultaneously, vital signs such as including heart rate, blood pressure, respiratory rate, and oxygen saturation will be continuously monitored.

#### Control group interventions

Patients in the control group will receive comprehensive conventional treatment, consisting of four main components: respiratory exercise, pharmacotherapy, airway management and positional management.

Respiratory function exercises will be conducted for 15 min twice daily at fixed time intervals (8:00–9:00 a.m. and 4:00–5:00 p.m.). The regimen will include deep breathing exercises, thoracic mobility training, and sputum expectoration training. During deep breathing exercises, patients will be instructed to inhale slowly and deeply through a tracheotomy cannula over 3 s, hold their breath for 2 s, and exhale slowly over 4 s. Each session will consist of 10 repetitions per set, with three sets completed and a 1 min rest between sets. Patients will be guided by a therapist to perform expansion movements of the upper, middle, and lower thoracic segments, five times for each segment. Sputum clearance training will include postural drainage, mechanical vibration expectoration, and instruction in effective coughing techniques, with each method applied for 5 min.

The pharmacological treatment protocol will be consistent with that of the experimental group, with appropriate antibiotics selected based on patients’ clinical manifestations and sputum culture results. For patients with viscous sputum, nebulized salbutamol (2.5 mg) combined with ipratropium bromide (0.5 mg) will be administered three times daily, at 8:00 a.m., 12:00 p.m., and 8:00 p.m. In addition, airway humidification with 5–10 mL of saline will be performed every 4 h to prevent sputum crust formation.

Airway management will involve assessing the need for suctioning every 4–6 h, with suctioning performed as necessary. The inner cannula of the tracheotomy tube will be changed twice daily, at 8:00 a.m. and 6:00 p.m., under strict aseptic conditions. Tracheotomy care will include daily cleaning of the skin around the tracheotomy site with 0.9% saline, and monitoring for signs of infection.

Position management will involve repositioning every 2 h to avoid maintaining the same position for long periods of time. Progressive positioning and limb mobility exercises will be performed for 15 min, twice daily.

All routine treatments will be delivered by medical personnel who have received standardised training. During treatment, healthcare professionals will closely monitor patients’ vital signs and immediately discontinue the intervention if any discomfort occurs. During the study period, patients will be prohibited from engaging in any other form of respiratory muscle training, adding new medications that may influence respiratory muscle function (e.g., systemic corticosteroids, neuromuscular blockers, or long-acting sedatives), or participating in other clinical trials that could affect respiratory function.

#### Outcome measures

The timetable for enrolment, intervention, and assessment is presented in Table 1.

**Table 1:** SPIRIT schedule of enrollment, interventions and assessment.

Study phase	Screening	Baseline	Intervention period			Follow-up period		
Visit	V0	V1	V2	V3	V4	V5	V6	V7
Time window	−7d ~ 0d	−24 h	Week 1	Week 2	Week 3	±2 h	±1d	±7d
Basic information								
Informed consent	√							
Demographics	√							
Medical history	√							
Inclusion/exclusion	√							
Primary outcome								
Decannulation readiness^a^			√	√	√			
Decannulation outcome					√	√	√	√
Secondary outcomes								
Diaphragm function^b^		√			√			
Respiratory parameters^c^		√			√			
Clinical outcomes^d^					√	√	√	√
Quality of life assessment^e^		√			√			√
Safety assessment								
Adverse events		√	√	√	√	√	√	√

V0 (screening): within 7 days before enrollment; V1 (baseline): within 24 h pre-intervention; V2-V4 (intervention period): ±1 day; V5 (post-decannulation 72 h): ±2 h; V6 (post-decannulation 1 week): ±1 days; V7 (3 months follow-up): ±7 days.

a Decannulation preparation assessment will include: The amount of secretion is small and the patient is able to clear it effectively; Effective cough with a peak cough flow rate >160 L/min; no hypoxaemia (SpO₂ ≥ 92%), respiratory distress, or decrease in the level of consciousness during the 24 h consecutive tracheostomy tube occlusion test; Swallowing function is normal.

b Diaphragmatic function assessment will include: diaphragmatic mobility and diaphragmatic thickness.

c Respiratory function parameters include: MIP, FVC, PEF, FEV1.

d Clinical outcome evaluation will include: length of hospital stay, rate of respiratory-related complications, and rate of reintubation within 72 h of decannulation.

e Quality of life assessment includes: EQ-5D-5L Health-Related Quality of Life Questionnaire, The Breathing Difficulty Assessment (Borg Scale), and The Hospital Anxiety and Depression Scale (HADS).

#### Primary outcome

The primary outcome will be the decannulation success rate following the 3-week intervention. Successful decannulation will be defined as the absence of reintubation within 72 h following tracheostomy tube removal. The decannulation process will adhere to a strictly standardised protocol, with specific criteria as follows: minimal secretions with effective clearance; good cough function (peak cough flow >160 L/min); absence of hypoxaemia (SpO2 ≥ 92%), respiratory distress, or reduced level of consciousness during a 24 h continuous tracheostomy tube occlusion test; and normal swallowing function. The decannulation success rate will be calculated as the proportion of successful decannulation relative to the total number of cases treated and expressed as a percentage. This outcome will be assessed at the end of the 3-week intervention.

Decannulation failure will be defined as the occurrence of any of the following conditions within 72 h post-decannulation: severe respiratory distress (respiratory rate >35 breaths/min); persistent hypoxaemia (requiring FiO2 > 0.5 to maintain SpO2 ≥ 90%); hypercapnia (PaCO2 > 50 mmHg and pH < 7.30); or clinical need for reintubation.

To ensure objectivity and accuracy, decannulation decisions will be made jointly by at least two therapists and rehabilitation physicians who are blinded to patient group allocation. This double-blind assessment design minimizes bias and enhances the reliability of study outcomes.

#### Secondary outcomes

Secondary outcomes will include diaphragmatic function, respiratory parameters, clinical outcomes, and quality of life.

Diaphragm function will be evaluated using a portable ultrasound device (SII, Sonosound Medical Devices Co., Ltd.). Diaphragm excursion will be measured using M-mode ultrasound. Patients will be positioned semi-recumbently, and a trained sonographer will use a convex-array probe placed at the mid-axillary line beneath the right costal margin to obtain the maximal diaphragm excursion during quiet breathing. Each measurement will be repeated three times and averaged. Diaphragm thickness will be measured with B-mode ultrasound using a high-frequency linear-array probe placed along the anterior axillary line at the 8th to 9th intercostal space, the thickness will be assessed at end-expiration and end-inspiration. All measurements will be performed at baseline (pre-intervention) and after 3 weeks of intervention.

Respiratory parameters, including MIP, forced vital capacity (FVC), peak expiratory flow (PEF), and forced expiratory volume in one second (FEV1), will be assessed using a portable spirometer (X1, Seck Medical Devices Co., Ltd., Xiamen, China). The tracheostomy cuff is inflated to 25 cmH₂O with patients in a semi-recumbent position (45°). For MIP assessment, patients will perform five maximal inspiratory efforts from end-expiratory volume, with the highest value being recorded from three reproducible trials, each demonstrating <10% variation. For spirometric parameters (FVC/PEF/FEV1), patients will perform three forced expiratory maneuvers from total lung capacity with ≥30 s rests between trials, adhering to ATS/ERS standards. Specifically, FVC will use the highest value from ≥2 reproducible trials (<5% variation), PEF will take the highest peak flow from any valid trial, and FEV1 will use the highest value from ≥2 trials with acceptable start-of-test criteria. All measurements will be performed at baseline (pre-intervention) and after 3 weeks of intervention.

Clinical outcome indicators will include the length of hospital stay, the incidence of respiratory-related complications, and the rate of reintubation within 72 h following decannulation. Relevant data will be extracted from patients’ electronic medical records. Length of stay will be defined as the total number of days from study enrollment to hospital discharge. Respiratory-related complications will be defined to include common conditions such as pneumonia, atelectasis, and pneumothorax. All clinical outcome indicators will be recorded by a designated study physician.

Quality of life will be evaluated using three instruments: the EQ-5D-5L Health-Related Quality of Life Questionnaire, the Breathing Difficulty Assessment (Borg Scale), and the Hospital Anxiety and Depression Scale (HADS). The EQ-5D-5L assesses five dimensions of health status—mobility, self-care, usual activities, pain/discomfort, and anxiety/depression—each rated on five levels. It also includes a visual analogue scale (VAS) ranging from 0 to 100 to evaluate the patient’s overall health status. Dyspnoea will be measured using the Revised Borg Scale (0–10 points), administered at rest and following standardized activities (e.g., sit-to-stand test), to reflect exertional breathlessness during daily tasks. The Hospital Anxiety and Depression Scale (HADS) comprises 14 items divided into two subscales—anxiety and depression—each containing seven items to evaluate corresponding symptoms. For patients unable to complete the questionnaires independently, assistance will be provided by trained research personnel to ensure accuracy and completeness. Assessments will be conducted at baseline (pre-intervention), upon completion of the 3 week intervention, and at 3 months post-intervention.

In the present study, a respiratory therapist will assess each patient’s readiness for decannulation daily and will document the decannulation attempt process and its outcome in detail. Post-decannulation follow-up will include relevant assessments within 72 h and a clinic or telephone visit during the first week. All assessments will be conducted by healthcare professionals blinded to group assignments. Ultrasound measurements will be obtained by trained physicians using a standardised protocol; respiratory parameters will be measured by qualified respiratory therapists; and quality of life will be assessed by study nurses.

Data will be collected at the following time points: baseline within 24 h prior to the intervention, and final assessment at the conclusion of the 3 week intervention period. Additionally, follow-up will be conducted in the clinic or via telephone 3 months after the intervention. Any adverse events observed during the evaluations, including discomfort during respiratory measurements or abnormalities in ultrasound examinations, will be recorded in detail. To minimize bias, all follow-up assessments will be conducted by trained assessors blinded to group allocation. For patients lost to follow-up, the study team will make proactive attempts to contact them via phone or text to collect outcome data. If contact cannot be established after three consecutive attempts, the patient will be classified as lost to follow-up.

### Data management

The data collection process for this study will be as follows: First, the study data will be recorded in the Electronic Medical Record (EMR) system, and trained researchers will transcribe the eligible data from the EMR into the standardized electronic Case Report Form (eCRF) based on the inclusion criteria. A complete audit trail will be maintained for all data entry and modification operations to comply with the requirements of International Council for Harmonisation—Good Clinical Practice (ICH-GCP).

This study employs a three-tiered data quality control strategy: first, source data validation by comparing the consistency between entries in the EMR and the eCRF; second, real-time validation built into the system, which employs logic checking and range validation tools to promptly identify data inconsistencies; and third, cyclical quality assessment involving weekly data quality reports to monitor data status. A full-time data manager and statistician supervised data collection to ensure strict adherence to the study protocol and regulatory standards. Upon completion of patient follow-up, original data will be digitised and archived, and the eCRF retained until data lock.

In addition, An independent Data Safety Monitoring Board (DSMB) will be established, consisting of neurologists, respiratory therapists, and statisticians. The DSMB will perform biannual blinded reviews to assess data integrity, quality, and potential safety concerns, and will independently provide recommendations to the study leaders based on these findings, thereby ensuring the safety and scientific validity of the study.

### Statistical analysis

The effect of the intervention will be evaluated based on both intention-to-treat (ITT) and per-protocol (PP) principles. Between-group comparisons for the primary outcome (decannulation success rate) will be conducted using the chi-square test or Fisher’s exact test, along with calculation of relative risk (RR), absolute risk reduction (ARR), and number needed to treat (NNT). Multivariate logistic regression analysis will be used to adjust for prespecified covariates such as age, sex, and stroke type. Between-group comparisons for continuous secondary outcomes will be conducted using t-tests or Mann–Whitney U tests. Repeated measures data will be analyzed using mixed linear models. Categorical secondary outcomes will be analyzed using the same approach as for the primary outcome. Time-to-event outcomes will be analyzed using Kaplan–Meier survival analysis and Cox proportional hazards regression models.

Missing data will primarily be handled using mixed-effects models under the missing at random (MAR) assumption, with multiple imputation and complete case analysis conducted as sensitivity analyses. Prespecified subgroup analyses will include factors such as stroke type and age, with effect heterogeneity evaluated using interaction terms and controlled for multiple comparisons via the Benjamini-Hochberg method. Sensitivity analyses will also include comparisons between ITT and PP outcomes, comparisons of different statistical methods, and assessment of covariate selection strategies.

All analyses will be performed using R software (version 4.2.0), employing two-sided tests with a significance level of 0.05. The analysis will be conducted by an independent statistician, and the analysis plan was pre-specified before enrollment of the first patient to ensure reproducibility and transparency. Categorical variables will be presented as frequencies (percentages), and group comparisons will be conducted using the chi-square test or Fisher’s exact test. Continuous variables will be presented as mean ± standard deviation or median (interquartile range), depending on distributional characteristics. Group comparisons will be conducted using the t-test or Mann–Whitney U test. Safety analyses will include the incidence of adverse events and between-group comparisons.

### Ethics and dissemination

This study was reviewed and approved by the Medical Ethics Committee of Guangdong Sanjiu Brain Hospital (approval no. 2025-01-006), and was registered with the Chinese Clinical Trial Registry (registration no. ChiCTR2500097604). Throughout the study, the ethical principles of the Declaration of Helsinki and the relevant regulations outlined in the Measures for the Ethical Review of Biomedical Research Involving Human Subjects will be strictly adhered to.

At enrollment, all participants or their legal representatives provided written informed consent before participating in the study. During the informed consent process, the investigators clearly explained the study’s purpose, procedures and potential risks and benefits to the participants. It was specifically emphasized that participation was entirely voluntary, and participants retained the right to withdraw at any stage without prejudice to their access to standard medical care. To facilitate participant withdrawal, a simplified procedure was implemented, requiring only verbal or written notification to the investigators. The informed consent form was written in plain language and sufficient time was provided for patients and their families to ask questions and consider relevant matters. All medical procedures and testing costs related to the study were covered, ensuring no additional financial burden for the patients. Participants were also informed that, in the event of a study-related adverse event, appropriate compensation and medical treatment would be provided.

The results of this study will be submitted to peer-reviewed journals in accordance with the publication standards of the International Committee of Medical Journal Editors (ICMJE) and the CONSORT statement, to ensure transparency in the research process and outcomes. Provided that participant privacy is fully protected, the study data will be made openly accessible to the academic community via a secure platform, in accordance with relevant protocols, to promote scientific exchange and further research.

## Discussion

Rehabilitation of respiratory function in post-stroke patients with tracheostomy has been a key focus of clinical research. Previous studies (15, 16) have confirmed that IMT can improve respiratory muscle function and strength, enhance cough efficiency, and promote pulmonary function recovery. However, traditional IMT methods have several limitations. For example, threshold loading devices lack flexibility and cannot adjust loading intensity based on the patient’s lung volume (20); they lack real-time feedback mechanisms, which affects patient compliance (21); existing protocols insufficiently account for the specific pathophysiological characteristics of patients with neuromuscular disorders (22); and most traditional IMT devices use fixed resistance, making it difficult to accommodate the dynamic demands of the respiratory cycle (23). Currently, most studies on TFRL-IMT focus on mechanically ventilated or COPD populations, with relatively few systematic investigations in post-stroke tracheostomy patients. Therefore, this study designed a randomized controlled trial to evaluate the effects of TFRL-IMT on respiratory function in post-stroke tracheostomy patients.

This study will adopt a single-centre, assessor-blinded, parallel-group randomized controlled design, with stratified block randomization used to control for confounding variables such as age and stroke type. The TFRL-IMT technique is innovative in providing a flow resistance load that decreases progressively with increasing lung volume, thereby ensuring balanced load distribution throughout the respiratory cycle (24, 25). A key strength of the study design is the establishment of a comprehensive assessment system including objective physiological indicators, clinical outcomes, and quality of life measures. The electronic training device employed in the study featured real-time data feedback, which can accurately quantify the training parameters and improve patient training compliance (25). To accommodate the characteristics of this specific population of post-stroke tracheostomy patients, the training load was dynamically adjusted based on the patient’s actual MIP percentage, thereby addressing the shortcomings of traditional methods in individualized design. Additionally, the study systematized the decannulation decision-making process and used a blinded assessment design to reduce assessment bias, thereby providing high-quality evidence for respiratory rehabilitation in post-stroke tracheostomy patients.

This study considers the potential mechanisms by which TFRL-IMT improves clinical outcomes as follows. The electronically controlled resistance valve system delivers a flow resistance load that varies with lung volume and conforms to the pressure–volume relationship of the respiratory muscles (24, 25), thereby optimizing the training load, which ensures that the respiratory muscles receive appropriate stimulation at different levels of lung volume, and inducing comprehensive neuromuscular adaptations (23, 29). Compared with traditional methods, TFRL-IMT enables greater lung volume expansion and higher inspiratory flow rates at the same training intensity (23), thereby enhancing tolerance to higher training loads and increasing the total workload (25). This physiological advantage facilitates the enlargement of respiratory muscle fiber cross-sectional area (30) and improvement in oxidative capacity (31), thereby strengthening overall respiratory muscle function. Moreover, load parameters scientifically set according to respiratory physiology principles (30% of MIP) induce adaptive changes in respiratory muscles while minimizing the risk of overfatigue (32). From a neurophysiological perspective, regular inspiratory muscle training can reshape the central nervous system’s control mode of the respiratory muscles (33, 34), enhance motor unit recruitment efficiency (35, 36), and contribute to the reconstruction of respiratory neural circuits after stroke (37). Furthermore, enhanced diaphragm function improves alveolar ventilation and enhances coughing ability and reduces the risk of infection (38), while the optimisation of oxygenation status brought about by improved respiratory function can reduce the inflammatory response, which in turn indirectly promotes neurological function recovery (39).

This study has several limitations. First, the generalisability of the findings is limited by the single-centre design. Second, the relatively short follow-up period (3 months) may not be sufficient to fully evaluate the long-term effects of the intervention. Additionally, this study relied on specific electronic devices during implementation, which may somewhat limit the dissemination and application of the study methodology to primary medical institutions or resource-limited areas. Although strict inclusion and exclusion criteria were applied, the highly heterogeneity of stroke patients—including varied comorbidities, stroke types, and severity—may result in diverse patterns of respiratory dysfunction, potentially affecting the generalisability of the findings.

This study protocol outlines the design of an exploratory randomized controlled trial evaluating a novel, electronically controlled TFRL-IMT approach in post-stroke tracheostomy patients. If proven effective, this intervention could serve as a valuable adjunct to standard rehabilitation by enhancing respiratory muscle function to facilitate decannulation. The findings will provide high-quality evidence to guide respiratory rehabilitation strategies in this vulnerable population. Furthermore, the standardized protocol may support future multi-center studies and personalized training programs. A comprehensive assessment of physiological, clinical, and patient-reported outcomes will offer a holistic understanding of the intervention’s impact.
